# Suicide Ideation in Hispanic and Latino Family Caregivers: Daily Prevalence and Risk Factors

**DOI:** 10.1093/geroni/igaf122.4015

**Published:** 2025-12-31

**Authors:** Felipe Jain, Natashia Bibriescas, Loreli Alvarez, Frank Puga

**Affiliations:** Massachusetts General Hospital / Harvard Medical School, Boston, Massachusetts, United States; University of Alabama at Birmingham, Birmingham, Alabama, United States; The University of Alabama at Birmingham, Birmingham, Alabama, United States; University of Alabama at Birmingham, Birmingham, Alabama, United States

## Abstract

Family caregivers face elevated risk of suicide ideation (SI). Hispanic and Latino (H&L) family caregivers of people living with dementia experience high burden yet remain understudied regarding SI. In H&L caregivers, we examined SI prevalence and associated risk factors using data from the Nuestros Días (Our Days) Study. 178 H&L family caregivers of people living with dementia (age: M = 56.3 (SD = 10.1), sex: 89.3% female) completed daily diary surveys (N = 3,237 observations) assessing SI over 21 days. Predictors, including depressive symptoms, coping strategies, emotion regulation, perceived stress, alcohol use, global health, and social isolation were assessed at baseline. Caregivers completed four questions daily to assess passive or active SI for 21 days (mean completion 18.2 (SD = 3.61) days). A stepwise zero-inflated Poisson regression, controlling for differences in the number of days observed with an offset, was used to predict total days of reported SI. 27.0% of participants (N = 48) reported passive or active SI on at least 1 day. Of these participants, the median number of days with SI was 2 (interquartile range 1 to 10.25). The final predictive model for days of SI included depression, emotional suppression, dysfunctional coping, emotion-focused coping, and social isolation (R2=.77, p<.001). Emotion-focused coping (sIRR=.637, 95% CI [0.534, 0.760], p<.0001) was significantly associated with fewer days of reported SI while social isolation (sIRR=2.337, 95% CI [1.680, 3.251], p<.0001) was significantly associated with more days of SI. In conclusion, SI is common among H&L family caregivers. Therapies that target improving emotion-based coping strategies and reducing social isolation might reduce SI.

